# Controlled Release of Nor-β-lapachone by PLGA Microparticles: A Strategy for Improving Cytotoxicity against Prostate Cancer Cells

**DOI:** 10.3390/molecules21070873

**Published:** 2016-07-02

**Authors:** Marcilia P. Costa, Anderson C. S. Feitosa, Fátima C. E. Oliveira, Bruno C. Cavalcanti, Eufrânio N. da Silva, Gleiston G. Dias, Francisco A. M. Sales, Bruno L. Sousa, Ito L. Barroso-Neto, Cláudia Pessoa, Ewerton W. S. Caetano, Stefano Di Fiore, Rainer Fischer, Luiz O. Ladeira, Valder N. Freire

**Affiliations:** 1 Department of Physiology and Pharmacology, Federal University of Ceará, 60430-270 Fortaleza, CE, Brazil; marciliapc@ufpi.edu.br (M.P.C.); acs_feitosa@yahoo.com.br (A.C.S.F.); cassiadefatima3006@gmail.com (F.C.E.O.); nunim_br@yahoo.com.br (B.C.C.); cpessoa@ufc.br (C.P.); 2 Department of Pharmacy, Federal University of Piauí, 64049-550 Teresina, PI, Brazil; 3 Department of Chemistry, Federal University of Minas Gerais, 31270-901 Belo Horizonte, MG, Brazil; eufranio@ufmg.br (E.N.S.J.); diasgg@yahoo.com.br (G.G.D.); 4 Department of Physics, Federal University of Ceará, 60455-760 Fortaleza, CE, Brazil; adilson.sales@gmail.com (F.A.M.S.); brunolopesdesousa@gmail.com (B.L.S.); 5 Department of Analytical Chemistry and Physical Chemistry, Federal University of Ceará, 60455-760 Fortaleza, CE, Brazil; itoliberato@gmail.com (I.L.B.-N.); vnffreire@gmail.com (V.N.F.); 6 Oswaldo Cruz Foundation (Fiocruz), 60180-900 Fortaleza, CE, Brazil; 7 Department of Secondary School and Teachers College, Federal Institute of Ceará, 60040-531 Fortaleza, CE, Brazil; 8 Fraunhofer Institute for Molecular Biology and Applied Ecology IME, 52074, Aachen, Germany; stefano.difiore@ime.fraunhofer.de (S.D.F.); rainer.fischer@ime.fraunhofer.de or fischer@molbiotech.rwth-aachen.de (R.F.); 9 Institute for Molecular Biotechnology, RWTH Aachen University, 52074 Aachen, Germany; 10 Department of Physics, Federal University of Minas Gerais, 31340-550 Belo Horizonte, MG, Brazil; loladeira@gmail.com

**Keywords:** nor-β-lapachone, lapachol, naphthoquinone, cancer, PLGA microcapsules, prostate

## Abstract

Prostate cancer is one of the most common malignant tumors in males and it has become a major worldwide public health problem. This study characterizes the encapsulation of Nor-β-lapachone (NβL) in poly(d,l-lactide-co-glycolide) (PLGA) microcapsules and evaluates the cytotoxicity of the resulting drug-loaded system against metastatic prostate cancer cells. The microcapsules presented appropriate morphological features and the presence of drug molecules in the microcapsules was confirmed by different methods. Spherical microcapsules with a size range of 1.03 ± 0.46 μm were produced with an encapsulation efficiency of approximately 19%. Classical molecular dynamics calculations provided an estimate of the typical adsorption energies of NβL on PLGA. Finally, the cytotoxic activity of NβL against PC3M human prostate cancer cells was demonstrated to be significantly enhanced when delivered by PLGA microcapsules in comparison with the free drug.

## 1. Introduction

Cancer is a progressive disease characterized by abnormal cell growth in which cells lose control over proliferation, overcome senescence, and become immortal and eventually malignant [1,2,3]. The molecular basis of cancer involves a combination of gain-of-function mutations in oncogenes and loss-of-function mutations in tumor-suppressor genes, often combined with epigenetic effects on gene expression [3]. Cancer is the leading cause of death worldwide [4], and death is generally caused by critical organ failure as the tumor cells spread by metastasis and seed secondary tumors [5].

Prostate cancer (PCa) is one of the most common malignant tumors in males [6], and it has become a major worldwide public health problem, reflecting high levels of morbidity and mortality [7]. The etiology of PCa, its incidence among major ethnic groups, and the molecular basis of the disease remain poorly understood. However, several factors are known to increase the risk of PCa, including senescence, family history, steroid hormone levels, pollutants, smoking, poor diet, as well as geographical and ethnic factors [7,8].

PCa shows a range of clinical behaviors, from slow-growing neoplasia to aggressively metastatic tumors [9]. There is currently no cure for metastatic PCa, and most patients have a poor prognosis once the disease reaches this stage [10,11]. Although several mutations can be used as markers of disease progression and therapeutic progress [10], a better understanding of the molecular basis underlying this disease is necessary in order to develop more effective treatments, as well as to decrease side effects [11].

Numerous plant-derived drugs, such as quinones, are known to possess promising anti-cancer activity, which could be explored for developing novel therapeutic strategies against PCa [12]. Quinones are the conjugated carbonyl groups in six-membered rings. Benzoquinones, naphthoquinones and anthraquinones are structurally related to benzene, naphthalene and anthracene, respectively. Quinones have been associated with different anti-cancer, anti-bacterial, anti-fungal, and trypanocidal activities [13], and their cytotoxicity may reflect a number of underlying mechanisms including redox cycling, leading to the production of reactive oxygen species (ROS) and the alkylation of cellular nucleophiles, prompting covalent binding with proteins and/or DNA [14,15,16]. In animal models, quinones may cause several harmful effects, including acute cytotoxicity, immunotoxicity and carcinogenesis [14]. Nevertheless, quinones are the second main class of anti-cancer drugs for clinical use, including several compounds such as dactinomycin, daunorubicin, doxorubicin, idarubicin, mitomycin-C and mitoxantrone [15,16,17,18,19].

**Figure 1:**
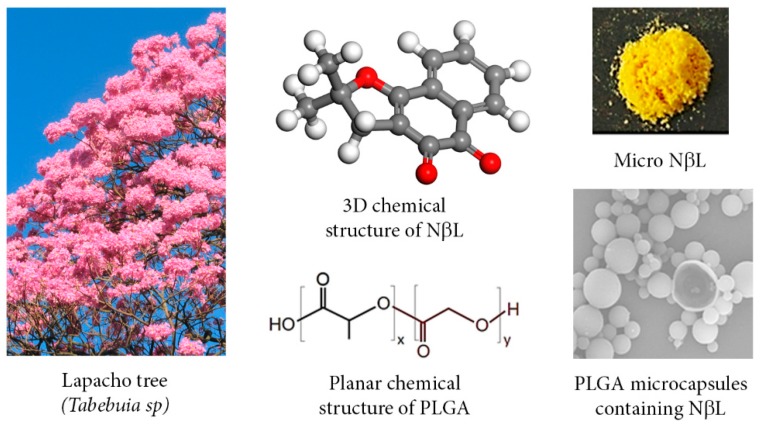
Naturally occurring naphthoquinone compounds are isolated from the lapacho tree (*Tabebuia* sp.). Nor-β-lapachone (NβL), a derivative prepared by the cyclization of nor-lapachol, was encapsulated in PLGA microcapsules by emulsification/solvent extraction. NβL-loaded PLGA microcapsules appear as a lyophilized orange powder, and SEM images (magnification ×5000) show the particle morphology.

Lapachol is a naturally occurring naphthoquinone isolated from the heartwood of species belonging to the *Bignoniaceae* family (*Tapebuia* sp.) (Figure 1), which along with its derivatives have been extensively investigated for the treatment of cancer [20]. Within this group, β-lapachone, one of the most widely-studied naphthoquinones, has presented a potent cytotoxic activity against several human cancer cell lines [21,22], requiring for its bioactivation the enzyme NAD(P)H:quinone oxidoreductase 1 (NQO1). Nevertheless, in view of the high liposolubility and non-specific distribution of β-lapachone, which have restricted its clinical use, different formulations for drug delivery systems have been proposed [23], including, polymer micelles [24], gold nanoparticles and cyclodextrin complexes within liposomes [25,26]. Several research groups have synthesized and investigated novel lapachone analogs exhibiting higher efficacies and less toxicity than β-lapachone [16,27].

**Scheme 1:**
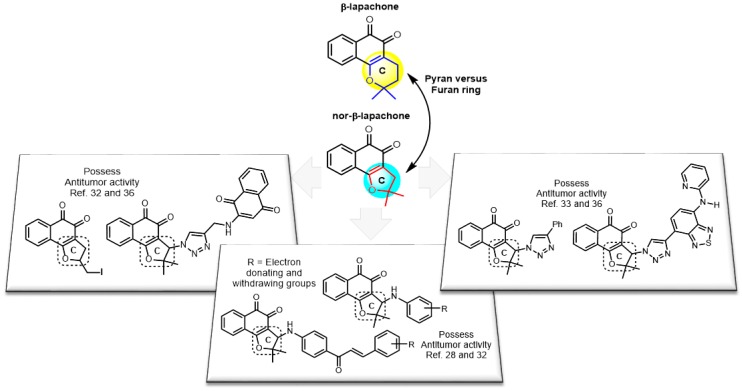
Structural modifications of the prototype nor-β-lapachone.

Nor-β-lapachone (NβL), 2,2-dimethyl-2,3-dihydronaphtho[1,2-*b*]furan-4,5-dione (Figure 1), is a lapachone derivative synthesized by the cyclization of nor-lapachol. It is considered an important anti-cancer prototype, and has been evaluated previously by our group against several cancer cell lines [22,28]. Previous reports have attested for NβL a higher cytotoxicity towards tumor cells when compared to healthy tissues [29], while also presenting important biochemical, hematological and histological toxic effects in Wistar rats [30]. In the last few years, our group described important strategies for preparing new potent antitumor compounds. Redox center, A- and C-ring modifications of naphthoquinone prototypes, such as, for instance, β-lapachone and NβL, were the subject of a study by us [13,31]. We have discovered several nor-β-lapachone derivatives with highlighted antitumor activity (Scheme 1) [28,32,33,34,35,36].

Currently, strategies for cancer treatment based on controlled delivery systems are able to optimize the therapeutic effect of drugs and reduce toxic side effects [37,38,39,40,41,42,43,44]. In this sense, nanoparticles and microcapsules are important vehicles for drug delivery [45]. Microcapsules (1–250 μm diameter) may be prepared through a variety of techniques [46], carrying larger drug loads than nanoparticles, as well as allowing a better control over sustained release profiles [47]. Based on this fact, several anticancer agents have been encapsulated in poly(d,l-lactide-co-glycolide) (PLGA) microcapsules (Figure 1) [48,49,50]. PLGA is a suitable option for developing controlled-release delivery devices due to its biocompatibility, biodegradability and the gradual release of drugs over a long period of time [39,46].

Here we present a PLGA-microencapsulated nor-β-lapachone formulation prepared by the emulsification/solvent evaporation method, which reduces the drug liposolubility and enables subsequent in vivo studies. Microcapsules were characterized by absorbance, Raman and infrared spectroscopy, as well as Fourier transform and differential scanning calorimetry (DSC). The, encapsulation efficiency, drug loading and in vitro release kinetics were determined using the spectroscopy methods listed above, whereas the interaction energy between NβL and the PLGA’s surface was estimated using classical molecular dynamics and annealing [51,52]. Scanning electron microscopy (SEM) and transmission electron microscopy (TEM) were used to evaluate the surface morphology and size distribution of the microcapsules, while the surface charge was determined by measuring the zeta potential. The in vitro cytotoxicity of the NβL-loaded microcapsules was investigated through cellular viability assays against several human prostate cancer cell lines, using empty PLGA microcapsules and a free NβL drug as controls. As discussed above, the antitumor activity of β-lapachone (quinone from lapachol group) was improved by the preparation of microparticles for use in controlled release. To the best of our knowledge, this is the first study involving controlled release of nor-β-lapachone microparticles and it represents a new strategy to improve the antitumor activity of this important naphthoquinone.

## 2. Results and Discussion

**Figure 2:**
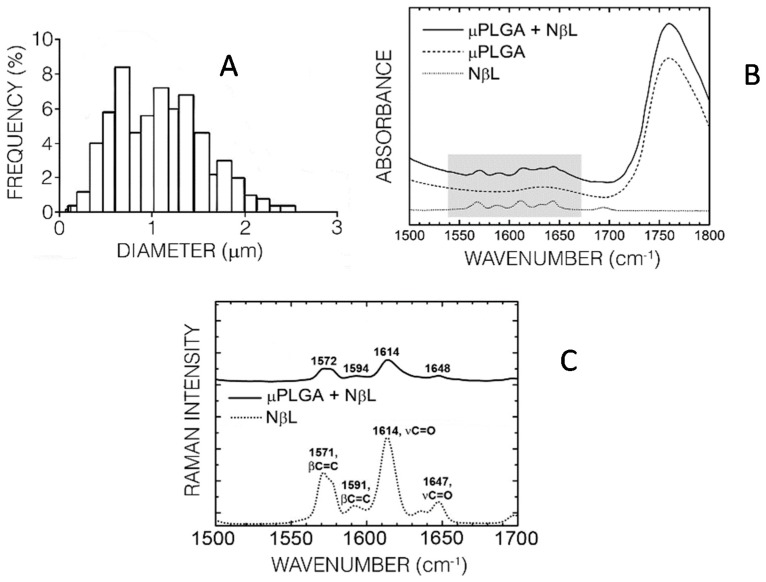
Characterization of the NβL-loaded PLGA microcapsules. (**A**) Size distribution of 500 microcapsules; (**B**,**C**) Respectively, infrared and Raman spectra of pure NβL (dotted line), empty PLGA microcapsules (dashed line) and NβL-loaded PLGA microcapsules (black line). The shading in the IR absorption spectrum highlights the wavenumber region depicted in the Raman plot. Raman band wavenumbers and their corresponding normal mode assignments are depicted as well.

PLGA microcapsule formulations were prepared by the emulsion solvent evaporation method, and the simple emulsion method was the most satisfactory for the preparation of NβL-loaded microcapsules. Briefly, NβL was dissolved in a polymeric solution of PLGA in dichloromethane and emulsified with aqueous PVA solution (2 min/11,000 rpm). In the process, stirring was performed for another four hours after the emulsification to evaporate the dichloromethane and produce a suspension of microparticles which were recovered by centrifugation and washed twice with deionized water. The best formulation was generated using PLGA 50:50 (100 mg), NβL (5 mg), 2% PVA solution (25 mL) and dichloromethane (10 mL). SEM and TEM analysis confirmed that the formulated microcapsules had appropriate morphological characteristics, such as a regular spherical shape and a smooth nonporous surface. The absence of drug crystals or clusters and the characteristic hollow structure of microcapsules were also confirmed. A sample of 100 NβL-loaded PLGA microcapsules exhibited a bimodal size distribution with average diameters of 1.03 ± 0.46 μm S.D. (standard deviation, Figure 2a). The most frequent diameter was 0.68 μm (8.4%), followed by 1.10 μm (7.2%). The largest observed diameter was 2.46 μm (0.39%) and the smallest was 0.14 μm (0.39%), with a mass median diameter close to 6 μm as estimated by using the Hatch-Choate equation. The calculated zeta potential of −23.4 ± 0.35 mV reflects the presence of NβL on the particle surface, which was also confirmed by infrared (Figure 2b) and Raman spectroscopy (Figure 2c). The last-revealed four peaks at 1572, 1594, 1614 and 1648 cm^−1^ in the spectrum represented the NβL-loaded microcapsules which were absent in the spectrum for the empty particles (not shown in Figure 2). These Raman bands were related to the peaks at 1571, 1591, 1614 and 1647 cm^−1^ observed in the pure NβL spectrum. The first two modes (1571, 1591 cm^−1^) can be assigned to benzene ring breathing modes where two C=C bonds on opposite sides of the hexagonal benzene ring perform symmetric stretching motions. The 1614 and 1647 cm^−1^ normal modes can be assigned to C=O bonds stretching in the naphthoquinone region of NβL. The FT-IR spectrum of the PLGA microcapsules loaded with NβL follows closely the spectrum of the unloaded microparticles, exhibiting a series of absorption bands between 490 and 3380 cm^−1^. In the region between 1500 and 1670 cm^−1^ one can see a set of characteristic peaks of NβL which are also present in the NβL-loaded PLGA microcapsules, but which do not appear in the empty formulation (Figure 2b).

**Figure 3:**
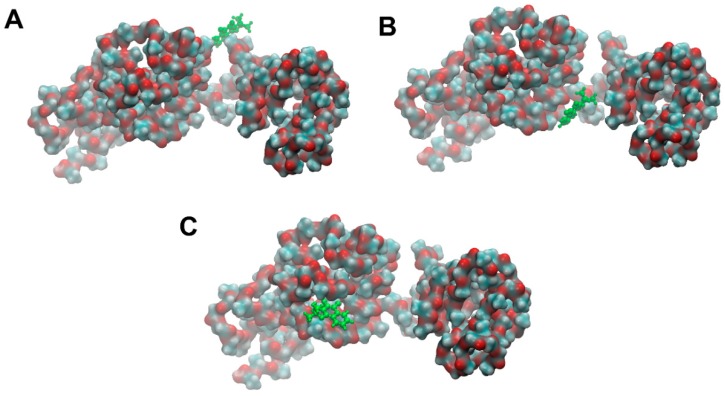
Classical molecular dynamics simulation of a single NβL molecule adsorbed on a PLGA microcapsule surface. (**A**) Structure with smallest binding energy; (**B**) Configuration with intermediate adsorption energy; (**C**) Adsorption geometry with largest binding energy.

A PLGA chain containing 100 monomers of lactic acid and 100 monomers of glycolic acid in a random sequence was optimized using classical annealing, producing a highly irregular polymer consisting of two asymmetric clusters connected by a small polymer bridge. A few indentations, cavities and clefts can be seen between these clusters where a single NβL molecule can be docked. Using Monte Carlo calculations, the surface of the annealed polymer was probed and 50 adsorption sites were found with adsorption energies ranging from −9.85 kcal·mol^−1^ (indicating physisorption or weak attractive interaction) to −31.5 kcal·mol^−1^ (indicating chemisorption or strong attractive interaction). This energy range of adsorption geometries provides some insight into the drug loading and entrapment efficiency as well as the initial phase of drug release (burst release, see below) by the microcapsules. Classical and quantum molecular dynamics simulations, on the other hand, can in principle be used to predict the effect of water solvation and pH on the process of drug release from the surface of PLGA microcapsules and nanoparticles. In Figure 3, three adsorption configurations are presented in increasing order of binding energy. Figure 3a depicts the structure with the smallest binding energy (−9.85 kcal·mol^−1^). Figure 3b reveals a configuration with intermediate adsorption energy (−18.1 kcal·mol^−1^), while Figure 3c is the adsorption geometry with the largest binding energy (−31.5 kcal·mol^−1^). In the last case, the NβL molecule appears inserted in a cleft on the larger cluster surface, with just one side not involved in interactions with the PLGA chain. We note that a recent work [53] on the release of drugs from mesoporous matrices revealed that this process becomes slower as the pore size is reduced. Computer simulations were carried out in order to understand this phenomenon, showing that the attraction of drug molecules to the walls of pores is stronger for smaller pores. The authors concluded that the drug release kinetics from mesoporous matrices must take into account the strength of the drug attraction by the mesoporous matrix. This motivates us to believe that there is a connection between drug-substrate binding energy values and the rate of drug release.

**Figure 4:**
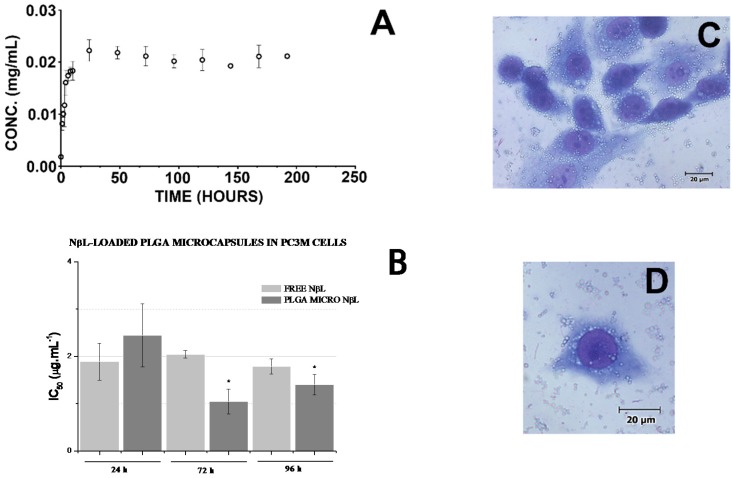
Drug release and in vitro assays. (**A**) Release profile of NβL from PLGA microcapsules under physiological conditions at 37 °C in PBS buffer (pH 7.4) (*n* = 3). Data are presented as mean values ± S.E.M. (standard error of the mean); (**B**) IC_50_ values for free and encapsulated NβL after 24, 72 and 96 h incubation with PC3M human prostate cancer cells. Data are presented as mean values ± S.E.M. from two independent experiments performed in duplicate (*n* = 4). * Significant difference from the PLGA micro NβL (24 h) group (*p* < 0.05) according to ANOVA Tukey’s test; (**C**,**D**) Microscopic analysis of hematoxylin/eosin-stained PC3M cells after 1 h of treatment with PLGA microcapsules containing NβL. The cells were analyzed by light microscopy (400×).

The DSC trace of the pure NβL showed a sharp endothermic peak at 192.04 °C, which corresponds to its melting point. The thermal gravimetric analysis (TGA) of the PLGA microcapsules containing NβL showed a mass gain due to decomposition. At the same time, thermal analysis has established that the incorporation NβL changes the thermal properties of the microcapsules. The absence of the peak corresponding to the melting point of the pure NβL in the PLGA microcapsules containing NβL suggests that the drug was dissolved or molecularly dispersed within the polymer in an amorphous fashion. The formulation with a 1:20 drug/polymer ratio showed the maximum drug encapsulation efficiency, with a total drug loading of 1.19% ± 0.07%, and an encapsulation efficiency of 19.36% ± 1.15%. In vitro release experiments were performed by the dialysis method. Briefly, approximately 150 mg of NβL-loaded PLGA microparticles were put into a dialysis bag and suspended in 200 mL of release medium (PBS, pH 7.4). The suspensions were incubated in glass tubes in an incubation shaker under stirring at 100 rpm at 37 °C. Samples of the solutions were removed at predetermined time intervals and the concentration of NβL was analyzed using a spectrophotometer at 450 nm. Release profiles were calculated in terms of the cumulative releasing percentage of NβL (%) over the incubation time. The release profile of NβL from the PLGA microcapsules in this study occurred in two stages (initial burst release phase followed by a second gradual release phase). Thus, drug release from the microcapsules followed a biphasic profile, with an initial burst effect within 24 h (∼90%) followed by sustained release for an extended time period (Figure 4a). This burst release effect can be assigned to the release of adsorbed drug molecules, which results in greater initial drug diffusivity [54].

A MTT assay was used to evaluate the cytotoxic effect of the free and encapsulated forms of NβL against three prostate cancer cell lines. The IC_50_ data for PC3M cells are presented in Figure 4b. As discussed by Pérez-Sacau et al. [55], the compounds can be classified as highly active (IC_50_ < 1 μg·L^−1^), moderately active (1 μg·mL^−1^ < IC_50_ < 10 μg·mL^−1^), or inactive (10 μg·mL^−1^ > IC_50_). Therefore, the NβL-loaded PLGA microcapsules have demonstrated effective cytotoxic activity against all the cell lines with the inhibitory activity of NβL-loaded PLGA microcapsules against PC3M cells exceeding the cytotoxicity of free NβL (Figure 3b). After incubation for 24 h, the IC_50_ values of the free and encapsulated NβL formulations were 1.887 (1.54–2.31) and 2.442 (1.86–3.21) μg·mL^−1^, respectively. After 72 h, the corresponding values were 2.045 (1.971–2.122) and 1.046 (0.82–1.34) μg·mL^−1^, and after 96 h they were 1.787 (1.63–1.96) and 1.401 (1.20–1.64). The drug was mostly toxic towards PC3M cells within the first 24 h and no additional cytotoxicity was observed until 96 h, probably due to the reduction of the drug concentration in the medium. After incubation for 72 h, the IC_50_ values of the free and encapsulated NβL formulations were significantly different according to ANOVA Tukey’s test. We suggest that in 72 h, the activity of the NβL-loaded PLGA microcapsules was increased because of the higher concentration of the drug within the cells. It was not observed any inhibitory effect of empty PLGA microcapsules over the proliferation of any cell line even at the maximum concentration of 10 μM (2.28 μg·mL^−1^). Microscopy revealed that the microcapsules adhered to the cell surface and were taken up by phagocytosis after a few minutes (Figure 4c). Pure NβL and PLGA microcapsules containing NβL did not present proliferative inhibition at the maximum concentration of 2.28 μg·mL^−1^ (10 μM) on PBMC.

## 4. Materials and Methods

Lapachol (2-hydroxy-3-(3′-methyl-2′-butenyl)-1,4-naphthoquinone) was extracted from the heartwood of *Tabebuia* sp. (Tecoma). Initially, a saturated aqueous sodium carbonate solution was added to the sawdust of ipê After the formation of the lapachol sodium salt, hydrochloric acid was added, allowing the precipitation of lapachol. After filtration, a yellow solid was obtained. This solid was purified by a series of recrystallizations with appropriate solvents. Nor-lapachol was prepared from lapachol by Hooker oxidation reaction [56]. Nor-β-lapachone was prepared by acid catalyzed cyclization from nor-lapachol as previously described [57]. The preparation and characterization of NβL-loaded PLGA microcapsules was discussed above for better understanding of the reader. PLGA microparticles nor-β-lapachone formulation was prepared by the emulsification in Ultra-Tturrax T25, IKA, Germany. The scanning electron microscope JEOL JSM 6360 LV (JEOL Europe SA, Croissy-sur-Seine, France) was used to carry out the measurements. The zeta potential of the PLGA microparticles was determined using Zetasizer Nano ZS (Malvern Instruments Ltd, Worcestershire, UK). Experimental measurements of the UV absorption spectra were carried out using the Varian Cary 5000 UV-visible NIR spectrophotometer (Varian Inc., Cary, NC, USA); all measurements were done on pellets made from KBr mixed with the sample powder. FT-IR spectra were all performed in a FTLA 2000 series laboratory instrument (ABB Bomem Inc., Québec City, QC, Canada); all spectra were run on a KBr pellet (100 mg, 1 wt %) and collected over 40 scans at a resolution of 2 cm^−1^. The differential scanning calorimetry (DSC) were analyzed using a DSC-60 (Shimadzu Corporation, Kyoto, Japan) thermal analyzer; the samples were heated from 25 to 220 °C at a heating rate of 10 °C/min under azote atmosphere (50 mm/min). Thermogravimetric analysis (TGA) was performed using a DTG-60H (Shimadzu Corporation, Kyoto, Japan); the samples were heated from 25 to 700 °C at 10 °C/min (azote atmosphere) and compared. **T**he drug content and loading efficiency were determined in triplicate using a Varian Cary 5000 UV-visible NIR (Varian Inc., Cary, NC, USA) spectrophotometer. To extract the NβL, known weights (~2 mg) of the lyophilized microparticles were broken with a mixture of dichloromethane and methanol (3:2) under ultrasound agitation for 30 min to ensure the complete polymer dissolution and release of the drug. The solubilized drug was quantified at 450 nm. In in vitro release tests were used an incubation shaker Cientec, CT712RTN (Cientec, SP, Brazil) and a DTX880 Multimode Detector (Beckman Coulter Inc., Pesadena, CA, USA) spectrophotometer. The human cell lines used in this work were HL-60 (leukemia), OVCAR-8 (ovarian), HCT-116 (colon), DU145 (prostate), PC3M (prostate) and PC3 (prostate), all obtained from the National Cancer Institute (Bethesda, MD, USA). The cells were maintained in RPMI 1640 medium supplemented with 10% fetal bovine serum, 100 U mL^−1^ penicillin, and 100 μg mL^−1^ streptomycin at 37 °C with 5% CO_2_ atmosphere. The cell viability was determined by the reduction of a yellow dye 3-(4,5-dimethyl-2-thiazolyl)2,5-diphenyl-2*H*-tetrazolium bromide (MTT) to a blue formazan product. Briefly, the cells were distributed in 96-well plates (OVCAR-8, DU145, PC3M and PC3 for 0.1 × 10^6^ cells/well in 100 μL of medium, 0.7 × 10^5^ cells/well for HCT-116 in 100 μL of medium and HL-60 for 0.3 × 10^6^ cells/well in 100 μL of medium). After 24 h, NβL dissolved in DMSO, empty PLGA microparticles and NβL-loaded PLGA microparticles suspended in PBS pH 7.4 at concentrations in the 1–10 μM range were added to each well. Doxorubicin was used as positive control (0.02–8.6 μM). Control groups received the same amount of DMSO. After 72 h of incubation, the plates were centrifuged and the medium was replaced with a fresh medium (150 μL) containing MTT (0.5 mg.mL^−1^). Only the prostate cancer cell lines (DU145, PC3M and PC3) were evaluated three times (within 24, 72 and 96 h). Three hours later, the plates were centrifuged, and the MTT formazan product was dissolved in 150 μL DMSO. The absorbance was measured using a multiplate reader (DTX880 Multimode Detector, Beckman Coulter Inc., Pesadena, CA, USA). Adherence of PLGA microcapsules was observed using an optic microscope (Olympus, Tokyo, Japan).

## 5. Conclusions 

The present study was designed to develop and characterize NβL-loaded PLGA microcapsules and to determine their cytotoxic activity against three human prostate cancer cell lines. Microcapsules obtained by emulsion solvent evaporation showed appropriate morphological features and a biphasic drug release profile. Drug loading was confirmed by spectroscopy, and classical molecular dynamics simulations indicated that NβL can be adsorbed on the surface of PLGA microcapsules with binding energies as large as −32 kcal mol^−1^ (chemisorption), contributing to the initial phase of the drug release process. Despite the relatively low encapsulation efficiency, the NβL-loaded PLGA microcapsules showed cytotoxic activity against all three cell lines, and were more cytotoxic than the free drug when tested against PC3M cells. Based on these data, we propose PLGA microcapsules containing NβL as a promising drug delivery system, suitable for in vivo model analysis of prostate cancer.

## Abbreviations

The following abbreviations are used in this manuscript:

NβL: Nor-β-lapachone
PLGA: Poly(d,l-lactide-co-glycolide)
PCa: Prostate cancer
ROS: Reactive oxygen species
DNA: Deoxyribonucleic acid
NQO1: NAD(P)H:quinone oxidoreductase 1
DSC: Differential scanning calorimetry
SEM: Scanning electron microscopy
TEM: Transmission electron microscopy
